# Survey analysis to determine the impact of evidence informed practice education upon East Asian medicine faculty clinical instruction and students’ skills, knowledge, attitudes and behaviors within a master’s degree program

**DOI:** 10.1186/s12909-021-02690-z

**Published:** 2021-05-04

**Authors:** Belinda J. Anderson, Saikaew Dudla, Paul R. Marantz, Benjamin E. Kligler, Brent D. Leininger, Roni Evans

**Affiliations:** 1 Pace University, College of Health Professions, 163 Williams St, New York, NY 10038 USA; 2 Pacific College of Health and Science, 110 William St, New York, NY 10038 USA; 3 Albert Einstein College of Medicine, 1300 Morris Park Ave, Bronx, NY 10461 USA; 4 United States Department of Veterans Affairs, Washington, D.C., USA; 5 University of Minnesota, Minneapolis, MN 55455 USA

**Keywords:** Evidence informed practice, East Asian medicine, Education, Acupuncture

## Abstract

**Background:**

Between 2013 and 2018 Pacific College of Health and Science (formerly Pacific College of Oriental Medicine) trained faculty and developed curriculum in evidence informed practice (EIP), with support from a grant from the National Institutes of Health (NIH). A three-credit (45 h) Foundations of EIP course, and online EIP learning modules (developed as part of a previous NIH R25 award), were used for faculty and student training. In addition, EIP was incorporated into 73% of the East Asian medicine degree program. Clinical integration of EIP in the College clinic was enhanced by improving access to reference sources, including additional EIP-related questions to the patient intake forms, requiring the use of a patient-centered outcome instrument, and assessing students’ clinical EIP competencies.

**Methods:**

Master’s degree students’ self-reported EIP skills, knowledge, attitudes and behaviors were assessed before and after taking the Foundations of EIP course using a 17-question paper-based survey with an additional open-ended comments section. The survey was administered in 29 courses across all three Pacific College campuses. Clinical faculty self-reported EIP instruction, focusing on the EIP content and instructional approaches that were utilized, was evaluated on the New York City campus using a paper-based survey before and after changes were made to enhance the clinical integration of EIP.

**Results:**

A total of 1181 completed EIP-course surveys consisting of 657 pre-EIP course surveys and 524 post-EIP course surveys were analyzed. There was a statistically significant improvement in students’ EIP skills, knowledge and behaviors after completing the EIP course. Students’ perception of the importance of EIP was high before and after the EIP course. Little change in Faculty’s EIP-related clinical instruction was evident following the EIP-related changes that were made to the Clinic.

**Conclusion:**

Our study suggests that the three-credit (45 h) EIP course was effective at improving the EIP skills, knowledge and behaviors of this group of East Asian medicine students who were undertaking a master’s degree that qualified them for licensure in acupuncture in the US. These students also demonstrated a high level of recognition for the importance of research and EIP both before and after the course. Training faculty clinical supervisors and providing greater access to evidence sources in the College clinic did not appear to increase EIP instructional activity.

## Introduction

Evidence-based medicine (EBM) is a foundational principle of healthcare education and practice. Defined as the “conscientious, explicit, and judicious use of current best evidence” along with clinical experience and patient preferences [1], EBM determines optimal healthcare treatment. Healthcare disciplines based on non-biomedical paradigms and theories, including many of the complementary and integrative health (CIH) therapies, have unique challenges in using EBM [2–5]. Of primary importance is the scientific evidence component of EBM. Although there is sufficient scientific evidence to suggest the use of several CIH approaches [6–8], concerns have been raised about external validity and generalizability of the outcomes and conclusions [9–14]. It has also led to a degree of skepticism by CIH practitioners [15–18]. In an effort to increase the research literacy of CIH disciplines and to enhance the use of research in CIH clinical practice, the National Institutes of Health (NIH) has targeted funding toward the development of research training for CIH practitioners.

Between 2013 and 2018, evidence informed practice (EIP) faculty training and curriculum development was undertaken at the Pacific College of Health and Science (formerly Pacific College of Oriental Medicine, PCOM). This project leveraged a previous NIH NCCIH R25 grant mechanism in which nine CIH institutions were funded to undertake EIP faculty training and curriculum development [19, 20]. Pacific College collaborated with faculty from Albert Einstein College of Medicine (Einstein) and Northwestern Health Sciences University (NWHSU) in this endeavor. NWHSU was one of the R25 grant recipients and undertook their EBM project in collaboration with the University of Minnesota (UMN).

As described in a previous publication [21] the approach for this project was to first train senior faculty, and then through the formation of a faculty led EIP curriculum committee, implement the curriculum revision. Faculty training was undertaken using online EIP modules [22] developed by NWHSU and UMN, and a three-credit (45 h) Foundations of EIP course. Both were based on the nine broad EIP competencies that were developed by NWHSU and UMN [23]. Ninety-one percent of the faculty and 97% of the clinical supervisors undertook the EIP training. EIP curriculum development consisted of developing the three-credit (45 h) Foundations of EIP course, and the introduction of EIP into 73% of the East Asian medicine master’s degree program, inclusive of 40 didactic courses and all (15) clinic shifts.

A master’s degree is the entry-level degree for East Asian medicine in the US, and the requirement for acquiring a state-based acupuncture licensure upon graduation and successful completion of board licensing exams. The scope of practice for licensed acupuncturists in most states of the US is restricted to East Asian medicine modalities. All accredited East Asian medicine colleges are required to have clinics where students fulfill the clinical requirements of their degree programs under the supervision of faculty (clinical supervisors) who have a license to practice acupuncture. Clinical education constitutes about one-third of the program hours. These clinics serve fee-paying public patients. EIP implementation in the College clinic was facilitated by providing better access to electronic reference sources, adding EIP related questions to patient intake forms, requiring the use of the Measure Yourself Medical Outcome Profile (MYMOP) [24, 25] outcome instrument, and including EIP competencies in students’ clinical assessment. Access to reference sources was improved by installing iPads in the Clinic consultation rooms where students and faculty clinical supervisors discuss patient cases and devise treatment strategies. The iPads facilitated access to PubMed and other biomedical internet sites, and were loaded with Chinese medicine text books and Apps.

Surveys were undertaken at the beginning of the project to assess faculty and students’ interest and support [26, 27], which showed that faculty and students had a high degree of interest and support for research. However, as students progressed through the 4 years of the degree program their interest in research declined, which is similar to trends observed at other institutions [28]. A more recent study of Canadian East Asian medicine students in an undergraduate diploma program did not show declines in research interest through the 3 years of their training [29]. However, this and the US studies, all showed that students had concerns about the appropriateness of scientific research models for investigating East Asian medicine (model validity). Such concerns stem from the recognition of paradigm and epistemological differences between East Asian medicine and biomedicine [6, 18, 29–31].

As part of this project the perspectives of Pacific College faculty and experienced acupuncturists about research and EBM were investigated and reported in two previous publications – a quantitative survey [26], and a later qualitative study [18]. Similar to students, faculty expressed concerns about model validity due to paradigm differences and the relevancy of research to clinical practice. They also raised the possibility of cooptation of East Asian medicine by biomedicine. The qualitative study focused on models for combining traditional, complementary and integrative medicine (TCIM) and biomedicine. Study participants, who were experienced acupuncturists (including some Pacific College faculty), showed a definite preference for a pluralistic model, which is characterized by different healthcare practices harmoniously and respectfully co-existing in a shared healthcare setting that permits each to operate with their own paradigms and methodologies [18, 30]. Their discussions also included concerns around model validity, cooptation and power imbalances between TCIM and biomedicine, and the importance of interprofessional education.

Of the nine recipients of the prior NIH NCCIH R25 grants that supported EBM faculty training and curriculum development, only two published their outcomes assessing changes in students’ EBM skills, knowledge, attitudes and behaviors in response to EBM training [32, 33]. Such outcomes are important for assessing the effectiveness of EBM training programs, their limitations, and for devising approaches for improvement. This study describes the outcomes of the EBM training undertaken at Pacific College. Pre- and post-course surveys were undertaken with students completing the 3-credit Foundations of EIP course, and clinical faculty were surveyed to assess their EIP-related clinical instruction. The learning, attitude and behavior changes resulting from the EBM training are discussed in relation to the unique challenges of undertaking EBM training with East Asian medicine students and faculty, and in other CIH disciplines.

## Methods and materials

### Quantitative EIP course survey

Participating students were taking the Foundations of EIP course face-to-face for three hours each week over 14 consecutive weeks as part of their master’s degree in East Asian medicine. These students were invited to complete a paper-based survey that was developed by researchers at NWHSU as part of their NCCIH R25 grant. This survey had not been psychometrically tested. The survey addressed perceived importance of EIP skills, confidence in EIP skills, EIP related behaviors and satisfaction. Questions related to skills were based on the nine EIP competencies that were developed as part of the parent R25 education program [23]. All of the questions in the survey are shown in Tables 1, 2 and 3. The surveys were administered to students at the beginning of the first class (pre-survey) and the beginning of the last class (post-survey) of each EIP course.

**Table 1:** Importance question responses from the pre- and post-course surveys (0 – not important to 10 – very important)

Question	Pre-EIP course^ *n* = 657	Post-EIP course^ *n* = 524	Difference^	Variability explained by	
				Student	Class
Ability to describe evidence-informed practice (EIP)	8.5(8.3 to 8.6)	8.7(8.5 to 8.8)	0.2(−0.03 to 0.4)	41%	3%
Ability to distinguish the strengths and weaknesses of different types of “evidence”	8.9(8.7 to 9.0)	9.1(9.0 to 9.2)	0.2(0.01 to 0.3)*	24%	1%
Ability to describe fundamental principles of research	8.4(8.2 to 8.5)	8.8(8.6 to 8.9)	0.4(0.1 to 0.5)*	36%	1%
Ability to efficiently find and retrieve research evidence that is relevant to your courses, assignments and clinical work	9.1(9.0 to 9.2)	9.2(9.1 to 9.4)	0.2(−0.01 to 0.3)	38%	1%
Ability to critically evaluate whether or not a research study is well-done	9.1(9.0 to 9.2)	9.2(9.1 to 9.4)	0.2(−0.003 to 0.3)	41%	2%
Ability to critically appraise the trustworthiness of other forms of evidence	8.9(8.8 to 9.0)	9.2(9.1 to 9.3)	0.3(0.08 to 0.4)*	43%	2%
Ability to integrate research evidence into coursework and assignments	8.6(8.5 to 8.7)	8.7(8.6 to 8.9)	0.1(−0.1 to 0.3)	44%	0%
Effectively apply relevant research evidence in clinical practice, in conjunction with patient preferences and clinical expertise	8.9(8.8 to 9.0)	9.0(8.9 to 9.2)	0.1(−0.03 to 0.2)	50%	1%
Effectively use research evidence to communicate with others	9.0(8.9 to 9.1)	9.0(8.9 to 9.1)	0.0(−0.2 to 0.1)	42%	1%
Identify ways to effectively participate in research in one’s field	8.2(8.0 to 8.4)	8.2(8.0 to 8.4)	0.0(−0.2 to 0.2)	58%	1%
Ability to customize a clinical encounter by taking into account individual patient preferences and presentation	9.0(8.9 to 9.1)	9.2(9.1 to 9.3)	0.2(0.04 to 0.4)*	36%	2%
Ability to recognize the strengths and limitations of experience (individual and professional)	9.1(9.0 to 9.2)	9.2(9.1 to 9.3)	0.1(−0.1 to 0.2)	35%	1%

^mean (confidence interval)

*p-value < 0.05

**Table 2:** Competency question responses from the pre- and post-course surveys (0 - not competent to 10 - very competent)

Question	Pre-EIP course^ *n* = 657	Post-EIP course^ *n* = 524	Difference^	Variability explained by	
				Student	Class
Ability to describe evidence-informed practice (EIP)	4.6 (4.5 to 4.8)	7.9 (7.7 to 8.1)	3.3 (3.1 to 3.5)*	15%	1%
Ability to distinguish the strengths and weaknesses of different types of “evidence”	4.9 (4.8 to 5.1)	8.0 (7.8 to 8.2)	3.1 (2.9 to 3.3)*	26%	1%
Ability to describe fundamental principles of research	4.7 (4.5 to 4.9)	7.9 (7.7 to 8.1)	3.2 (3.0 to 3.5)*	24%	2%
Ability to efficiently find and retrieve research evidence that is relevant to your courses, assignments and clinical work	5.7 (5.5 to 5.9)	8.5 (8.3 to 8.6)	2.7 (2.5 to 3.0)*	24%	2%
Ability to critically evaluate whether or not a research study is well-done	5.1 (5.0 to 5.3)	8.0 (7.8 to 8.2)	2.9 (2.6 to 3.1)*	31%	3%
Ability to critically appraise the trustworthiness of other forms of evidence	5.3 (5.1 to 5.4)	8.0 (7.8 to 8.2)	2.7 (2.5 to 3.0)*	37%	2%
Ability to integrate research evidence into coursework and assignments	5.6 (5.4 to 5.7)	8.1 (7.9 to 8.3)	2.5 (2.3 to 2.8)*	27%	2%
Effectively apply relevant research evidence in clinical practice, in conjunction with patient preferences and clinical expertise	4.9 (4.7 to 5.0)	7.9 (7.7 to 8.1)	3.0 (2.8 to 3.3)*	25%	2%
Effectively use research evidence to communicate with others	5.2 (5.0 to 5.4)	7.9 (7.8 to 8.1)	2.7 (2.5 to 3.0)*	30%	2%
Identify ways to effectively participate in research in one’s field	4.0 (3.9 to 4.2)	6.9 (6.7 to 7.1)	2.9 (2.6 to 3.1)*	44%	0%
Ability to customize a clinical encounter by taking into account individual patient preferences and presentation	5.1 (4.9 to 5.2)	8.1 (7.9 to 8.3)	3.0 (2.8 to 3.2)*	33%	1%
Ability to recognize the strengths and limitations of experience (individual and professional)	5.5(5.4 to 5.7)	8.1 (7.9 to 8.3)	2.5 (2.3 to 2.7)*	42%	1%

^mean (confidence interval)

*p-value < 0.05

**Table 3:** Behavior and satisfaction question responses from the pre- and post-course surveys

Questions	Pre-EIP course^ *n* = 657	Post-EIP course^ *n* = 524	Difference^	Variability explained by	
				Student	Class
**Behaviors** (1 to 4 scale with higher numbers indicating more frequent behavior 1 = <once/month; 4= > once/week)					
Discussed research information with others?	2.8 (2.7 to 2.8)	3.8 (3.7 to 3.9)	1.1 (0.9 to 1.2)*	42%	1%
Accessed a summary resource to keep up-to-date with research evidence in healthcare?	2.4 (2.3 to 2.5)	3.6 (3.5 to 3.7)	1.2 (1.1 to 1.3)*	15%	3%
Tried to find research evidence to answer a clinical question?	2.5 (2.4 to 2.6)	3.4 (3.3 to 3.5)	0.9 (0.8 to 1.0)*	40%	3%
Applied the evidence informed practice model (integration of patient presentation, clinical experience and research evidence) in clinical decision making?	1.9 (1.8 to 2.0)	3.1 (3.0 to 3.2)	1.1 (1.0 to 1.3)*	28%	2%
**Satisfaction** (0 – not satisfied to 10 – very satisfied)					
Overall, how satisfied are you with the quality and content of research-related coursework you have taken at PCOM to date?	5.6(5.5 to 5.8)	8.3(8.1 to 8.5)	2.6(2.4 to 2.9)*	18%	2%

^mean (confidence interval)

*p-value < 0.05

Pre- and post-surveys began with three coding questions designed to allow linking of the pre- and post-course participant responses. These questions were the first 2 letters of their Mother’s first name, day of birth month, and the last letter of their first name. Following the coding questions, the pre- and post-surveys had an identical 17 questions (Tables 1, 2 and 3). Post-surveys also had one additional question (question 18). The first 12 questions (Tables 1, 2 and 3) required the survey taker to respond to each question in two parts - A and B. Part A, asked ‘How important you think it is for you as a healthcare professional to have the following skills?’. Part B. queried ‘How you would rate yourself now in terms of these skills?’ Responses to part A assessed EIP attitudes and were a 0–10 Likert scale where 0 equaled ‘not important’ and 10 equaled ‘very important’. Responses to part B assessed EIP skills and knowledge and were a 0–10 Likert scale where 0 equaled ‘not competent’ and 10 equaled ‘very competent’. Questions 13–16 related to EIP behaviors, and used a 1–5 Likert scale where 1 equaled ‘never’, 2 equaled ‘less than once per month’, 3 equaled ‘1-2 times per month’, 4 equaled ‘3-4 times per month’, and 5 equaled ‘more than once per week’. Satisfaction was assessed in question 17 by asking ‘Overall, how satisfied are you with the quality and content of research-related coursework you have taken at PCOM to date?’ and had a 0–10 Likert scale response where 0 equaled ‘not satisfied’ and 10 equaled ‘very satisfied’. The final question 18 in the post-survey, asked about further research interest – ‘Would you like to work on a research study as part of your student experience?’ and had a yes/no response. The questions were followed by an open-ended comments section.

### Clinical supervisors’ survey

The Clinical Supervisors’ Survey was undertaken after these faculty had completed their EIP training [21]. Faculty who are clinical supervisors instruct students treating patients in the College clinic. The students perform acupuncture therapy treatments under the clinical supervisor’s acupuncture license. Surveys were completed before (pre) and after (post) changes were made to the clinical policies and procedures to facilitate the use of EIP in the clinic [21]. These changes included the provision of electronic access to reference sources, the addition of EIP related questions to the patient intake forms, the required use of an outcome instrument - the MYMOP [24, 25], and including EIP competencies in students’ clinical assessment. Both surveys had nine questions, and the post-survey had three additional questions. The survey questions were preceded by the statement – ‘Please indicate how often you do each of the following in the process of teaching your students on shift at the PCOM Clinic’. The responses to the questions were scored using a 1–4 Likert scale where 1 equaled ‘never’, 2 equaled ‘once or twice’, 3 equaled ‘in about half of my clinic shifts’, and 4 equaled ‘in almost all my clinic shifts’. These surveys had not been psychometrically tested, and no coding questions to allow linking of survey takers were included.

### Survey implementation

Use and implementation of these surveys was approved by the Einstein (IRB number 2014–3522) and Pacific College (protocol number 14–001) institutional review boards, and verbal informed consent was obtained from all participants. All methods were carried out in accordance with relevant guidelines and regulations.

The surveys were paper-based. Students completed the pre- and post-EIP course surveys in class. Clinical faculty were asked to complete the Clinical Supervisors’ surveys during a break in the clinical department meetings. For both students and faculty, survey participation was voluntary with no penalty for non-participation.

The pre- and post-EIP course surveys were administered in 29 Foundations of EIP courses. All on-ground Foundations of EIP courses were included during all 5 years of the EIP curriculum development project from 2014 till 2018. These surveys were undertaken on all three Pacific College campuses in NY, San Diego and Chicago. The Clinical Supervisors’ survey, conducted only on the NY campus, was undertaken in March 2017 (pre) and October 2018 (post) during the 4th and 5th years of the EIP curriculum development project, respectively.

### Statistical analyses of the quantitative EIP course survey

Changes in perceived importance of EIP skills, confidence in EIP skills, EIP behaviors, and satisfaction were assessed using linear mixed models with nested random effects to account for the correlated nature of the data. Linear mixed models have a number of advantages over repeated measures ANOVA for the analysis of paired or correlated data. One advantage is their ability to account for multi-level hierarchies within the data (i.e. repeated measures from students within classes within schools). Another important advantage is the ability to include pre- or post-course survey data from students missing one of the assessments, while still accounting for the matched nature of the data from students who completed both assessments. Timing of the survey (pre vs post) was included as a fixed effect in each model to estimate the mean difference between the pre- and post-survey as the main effect. We included a random intercept for student to account for the paired data from students completing both pre- and post-course surveys. A random intercept for class cohort was also included to capture the nested structure of the data and explore variation in effects across classes. A random effect for location was not considered due to the limited number of clusters available (*n* = 3). Location and year were included as fixed effects to control for differences across campuses and time. Confidence intervals for changes in perceived importance of EIP skills were constructed using bootstrap methods (1000 iterations) due to the skewed nature of their distributions. Importance of EIP skills were considered as a potential confounder for analyses of confidence in EIP skills; however, the relationship between importance of EIP skills and changes in confidence were very weak and were not included as covariates (absolute correlations < 0.12).

### Qualitative analysis of the open-ended EIP course survey question

Comments that were written in the open-ended comments section of the pre- and post-EIP course surveys were transcribed into a spread sheet document by SD. BA and SD read through the comments and met to discuss emerging themes. SD categorized the comments into 29 categories. BA read through these categories and condensed them into six themes. SD and BA evaluated and discussed these themes, and decided upon the final six themes presented in this paper.

### Statistical analysis of the clinical supervisors’ survey

For the Clinical Supervisors’ surveys, we assessed if changes in the frequency of EIP engagement in the clinic had occurred after changes were made to the clinic policies and procedures to facilitate the use of EIP in the clinic. A chi-square test was used to assess differences in the frequency of EIP engagement before and after these changes were made.

All quantitative analyses were conducted using Stata 13.1 [35].

## Results

### Quantitative EIP course survey

Survey data was derived from students in 29 Foundations of EIP courses – 17 on the NY campus, eight on the San Diego campus, and four on the Chicago campus. A total of 1181 completed EIP-course surveys consisting of 657 pre-EIP course surveys and 524 post-EIP course surveys were analyzed. The survey response rate among the various EIP courses was over 90%. An exact calculation of the response rate was not possible due to slight fluctuations in student numbers as a result of students dropping and adding the courses. Pre- and post-surveys were able to be matched using the three linking questions for 233 of the 657 (35%) pre-course surveys, and 233 of the 524 (44%) of the post-course surveys. Inability to match pre- and post-course surveys was due to inconsistent answers to the linking questions and in some cases illegible handwriting.

Changes in perceived importance of EIP skills, confidence in EIP skills, EIP behaviors, and satisfaction are shown in Table 1, 2 and 3 along with the amount of variability explained by class effects and repeated measures from students. Mean pre-EIP course scores for the importance of EIP skills were high and ranged from 8.2 to 9.1 (Table 1). There were minimal changes in the perceived importance of EIP skills following the course with the largest increase of only 0.4 on a 0–10 scale. Mean pre-EIP course scores for confidence in EIP skills were lower and ranged from 4.0 to 5.7 (Table 2). Confidence in EIP skills showed larger, more consistent improvements following the course relative to perceived importance, with mean changes ranging from 2.5 to 3.3 on a 0–10 scale. All improvements in confidence in EIP skills were statistically significant.

Mean pre-EIP course scores for EIP behaviors (Table 3) ranged from 1.9 to 2.8 on a 1–5 scale, which indicated students were, on average, engaging in the behaviors from less than once a month to 1–2 times per month. Increases in self-reported engagement in EIP behaviors ranged from 0.9 to 1.2 following the course. This corresponds to students engaging in the behaviors closer to 3–4 times per month. All four questions showed a statistically significant increase in EIP related behaviors. Overall satisfaction with research-related course work at the College (Table 3) was moderate prior to the course (5.6 on a 0–10 scale) and increased by 2.6 (95% CI 2.4 to 2.9) following the course.

Question 18 was only on the post-EIP course survey and asked about further research interest with a yes/no response. Of the 524 respondents 160 (31%) answered yes, 69 (13%) answered no, and 295 (56 %) did not answer this question.

Overall, class effects had little impact on the observed results and were only able to explain 3% or less of the variation of changes in perceived importance of EIP skills, confidence in EIP skills, EIP behaviors, and satisfaction (Tables 1, 2 and 3). The impact of repeated measures from students completing both pre- and post-course surveys was more pronounced, accounting for as little as 15% to as much as 58% of the variability in estimated changes (Tables 1, 2,and 3).

### Qualitative analysis of open-ended EIP course survey question

Table 4 lists the six themes that comments to the open-ended question on the pre- and post-EIP course surveys were categorized into. There was a total of 213 comments (91 pre- comments and 122 post-EIP course comments) that contained content that was relevant to the EIP course. Of the 657 pre- and 524 post-course surveys that were included in this analysis, the response rate for this open-ended question was 14% for the pre-course surveys and 23% for the post-course surveys. Comments often contained content that was categorized into more than one of the six themes. Therefore, the number of comments listed in Table 4 for the pre- and post-EIP course surveys exceeds the 213 individual comments.

**Table 4:** Theme analysis of EIP course open-ended question

Themes	Content	Number of comments^a^		
		Pre *n* = 91	Post *n* = 122	Total (%)^b^ *n* = 213
Satisfaction	class, instructor, education, enthusiasm to learn	18	75	93 (31)
Positive view of research	changed perspective of research, research is important - legitimizes the profession, interested in doing research, relevance and application of skills learned, would like more of this content	10	57	67 (22)
Dissatisfaction	length of the class, too challenging, not challenging enough, too late in the program, online modules, need more practice	12	36	48 (16)
Comparison to other courses in the degree program	other courses presented unscientific perspectives, prefer Chinese medicine to biomedicine, research not well integrated into other courses	39	2	41 (14)
Reference to prior learning	transfer student, little prior exposure to research, little clinical experience	24	5	29 (10)
Negative view of research	not interesting, not valuable, cooptation concerns, paradigm differences, irrelevancy to clinical practice	10	10	20 (7)

^a^Comments from individual respondents were frequently categorized into more than one theme. Therefore, the number of comments exceeds the number (n) of respondents

^b^Total comment percentages were calculated by dividing the total number of comments for each theme by the total number of comments that had been categorized into the six themes (298)

The majority (31%) of the comments were expressing satisfaction. Most of these were recorded post-course and focused on the class, instructor, education and learning experience. An example of a post-course comment was “Awesome class, very fundamental, & I think everyone should takes this course. Other classes should follow this example instructor.” The next most frequent theme (22%) were comments expressing a positive view of research. These included comments indicating that the course changed research perspectives, showed the value of research in legitimizing the profession, generated an interest in research, and imparted useful skills. Most of these comments appeared in the post-course surveys. Examples of comments for this theme were - “Excellent course, very informative; I’ve been able to apply methods learned in this class to other classes, as well as clinic”, and “Good class, I learned how to critically assess research in a presentable and systematic way, tailored to my specific needs (as a future practitioner).”

Sixteen percent of the comments were expressing dissatisfaction and were mainly in the post-course surveys. Dissatisfaction was with the length of the course, its level of challenge (both too much and too little), placement sequence in the program curriculum, use of online modules for part of the course, and the need to be more clinically experienced. Examples of comments were - “FEIP is a very important subject for health care professionals, however the actual class was intensely overcomplicated resulting in most of the class not caring about the subject matter”, and “This is very important class, but I think it was given on elementary level, not up to par with adults of masters level professionalism”.

In the pre-course surveys we had a predominance of comments comparing this course to others in the degree program. These comments mentioned that other courses the students had taken did not integrate research and presented unscientific perspectives, and a preference for East Asian medicine courses. Examples of this theme were - “I cannot name a class that places research-related coursework at more than 5% of total grade, and I do not value it personally at all”, and “I understand the validity of published work in the field but hope to see more of the classics involved & less of a westernized approach”.

There were two minor themes. Ten percent of comments referenced prior learning and were mainly in the pre-course comments. These commented on being a transfer student, and having little prior research and clinical experience. Seven percent of the comments expressed a negative view of research and were in both the pre- and post-course surveys.

### Clinical supervisors survey

Data from the Clinical Supervisors’ survey is shown in Tables 5 and 6. Eighteen pre-surveys and 28 post-surveys were completed representing 49 and 76% of the 37 supervisors, respectively. The range of responses to the nine identical questions in the pre/post surveys are shown in Table 5. The only question that showed a statistically significant change from the pre- to post-survey was in relation to asking students to cite articles, which increased in the post- survey. Activities that appeared to be taking place the most frequently were comparing and contrasting different approaches, identifying sources of information when teaching, and providing references to students. Responses to the three additional questions on the post-survey are shown in Table 6.

**Table 5:** Clinical Supervisors’ survey – responses to questions in the pre- and post-surveys

Questions	Pre *n* = 18	Post *n* = 28	Χ^**2**^	***P*** value
Have students cite articles, n (%)			10.7	0.01
*Never*	3 (17.6)	9 (34.6)		
*Once or twice*	14 (82.4)	9 (34.6)		
*In about half of my clinic shifts*	0 (0)	4 (15.4)		
*In almost all my clinic shifts*	0 (0)	4 (15.4)		
Discuss how to conduct a literature review, n (%)			2.5	0.46
*Never*	7 (41.2)	12 (44.4)		
*Once or twice*	9 (52.9)	10 (37)		
*In about half of my clinic shifts*	0 (0)	3 (11.1)		
*In almost all my clinic shifts*	1 (5.9)	2 (7.4)		
Compare and contrast scientific articles, n (%)			5.2	0.16
*Never*	4 (22.2)	6 (23.1)		
*Once or twice*	13 (72.2)	12 (46.2)		
*In about half of my clinic shifts*	0 (0)	5 (19.2)		
*In almost all my clinic shifts*	1 (5.6)	3 (11.5)		
Compare and contrast different approaches, n (%)			0.75	0.86
*Never*	1 (5.6)	1 (3.6)		
*Once or twice*	4 (22.2)	9 (32.1)		
*In about half of my clinic shifts*	6 (33.3)	7 (25)		
*In almost all my clinic shifts*	7 (38.9)	11 (39.3)		
Discuss strengths and weaknesses of studies, n (%)			4.1	0.25
*Never*	5 (29.4)	2 (7.4)		
*Once or twice*	6 (35.3)	10 (37)		
*In about half of my clinic shifts*	3 (17.6)	7 (25.9)		
*In almost all my clinic shifts*	3 (17.6)	8 (29.6)		
Identify source of information when teaching, n (%)			2.0	0.56
*Never*	1 (5.9)	1 (3.6)		
*Once or twice*	3 (17.6)	3 (10.7)		
*In about half of my clinic shifts*	7 (41.2)	8 (28.6)		
*In almost all my clinic shifts*	6 (35.3)	16 (57.1)		
Provide references to students, n (%)			0.15	0.93
*Never*	0 (0)	0 (0)		
*Once or twice*	3 (17.6)	6 (21.4)		
*In about half of my clinic shifts*	5 (29.4)	7 (25)		
*In almost all my clinic shifts*	9 (52.9)	15 (53.6)		
Have students provide references for treatment, n (%)			4.4	0.22
*Never*	2 (11.1)	4 (14.8)		
*Once or twice*	6 (33.3)	6 (22.2)		
*In about half of my clinic shifts*	8 (44.4)	7 (25.9)		
*In almost all my clinic shifts*	2 (11.1)	10 (37)		
Have students discuss ideas for research, n (%)			3.7	0.30
*Never*	4 (22.2)	5 (17.9)		
*Once or twice*	8 (44.4)	11 (39.3)		
*In about half of my clinic shifts*	6 (33.3)	7 (25)		
*In almost all my clinic shifts*	0 (0)	5 (17.9)

**Table 6:** Clinical Supervisors’ survey – additional questions in the post-survey

Questions	Post *n* = 28
Use the electronic resources yourself in the clinic consultation rooms, n (%)	
*Never*	5 (19.2)
*Once or twice*	5 (19.2)
*In about half of my clinic shifts*	5 (19.2)
*In almost all my clinic shifts*	11 (42.3)
Ask your students to use the electronic resources in the clinic consultation rooms, n (%)	
*Never*	4 (15.4)
*Once or twice*	5 (19.2)
*In about half of my clinic shifts*	4 (15.4)
*In almost all my clinic shifts*	13 (50)
Undertake or supervise a MYMOP evaluation, n (%)	
*Never*	9 (33.3)
*Once or twice*	6 (22.2)
*In about half of my clinic shifts*	8 (29.6)
*In almost all my clinic shifts*	4 (14.8)

## Discussion

Our study showed that Pacific College students had a high degree of recognition of the importance of EIP before taking the Foundations of EIP course, and that this remained high after completing the course. Responses to the open-ended survey question also suggest that the course may have engendered a positive view of research. Their self-rated EIP competence improved significantly after completing the course as did all EIP behaviors. After taking the EIP course students appeared to be discussing research every week, and their frequency of accessing summary sources and applying the EIP model increased from once per month to once every other week. Little variation between the various questions for both importance and competence was observed. The practice of EIP by the clinical supervisors was moderate, and was little changed after improvements for EIP engagement were made in the College clinic.

The trend of recognizing the importance of EIP and research has been shown in our and several other prior studies with both East Asian medicine students and faculty [18, 26–29]. In these studies, the importance of research was mainly with respect to those outside the profession – the public, referring providers, and insurance companies. Two of the three studies, at three different East Asian medicine colleges, also showed that students’ interest in research, and its perceived value to clinical practice, declined as they progressed through their training [26, 28]. This same trend has also been reported with chiropractic students [32].

This trend of valuing the importance of research for those outside the profession may be the reason why the students in this survey had a high degree of recognition of the importance of EIP both before and after taking the EIP course. In fact, comments in the open-ended survey question expressing a positive view of research were much more frequent in the post-course surveys, and only 7% of the pre- and post-course comments expressed a negative view of research. This same trend was seen in the study of Canadian East Asian medicine students where their perception of the importance of research did not diminish after taking a 30-h research literacy course [29]. This contrasts with the prior studies [26, 28], which showed that students’ perception of the importance of research to their clinical practice declined as they progressed through their training. An important difference between these studies is that in this study the perceived importance of research skills and knowledge was assessed before and after a single course over the period of a few months, whereas the previous studies [26, 28] assessed changes throughout a degree program over three to 4 years. In the latter, students likely had more exposure to the clinical practice of East Asian medicine, and therefore greater opportunity and experience to determine the differences between treatments administered in clinical trials and those performed in real-world clinical settings.

Another possible explanation for the difference between this and the Canadian study, compared with the previous studies, is the way in which research methodology has changed over the past decade. Clinical research in East Asian medicine started with efficacy trials and use of randomized placebo-controlled trials. Problems associated with the validity of this clinical trial methodology have been widely discussed in the literature for both TCIM therapies [29, 34, 36, 37] and biomedicine [38]. For example, the acupuncture treatments often are not representative of real-world practice; this, along with other study related issues (e.g. interpreting meaningful group differences, etc.) limit the capacity to inform clinical practice. The predominance of these types of trials has led to suspicion and disregard of research by East Asian medicine practitioners [15–18].

Recognition of trials that are more fastidious in nature, has led to the development of different research models, such as pragmatic clinical trials [10], comparative effectiveness trials, whole systems research [29], and mixed methods. Trials using these approaches are now beginning to become more popular in the research literature. The EIP course developed in this project [21], and that used in the Canadian study [29], included content on these new research models, along with discussions about the weaknesses of the randomized placebo-controlled trial design. This, combined with the presence of such trials showing up in students’ literature searches, may also explain why we did not see declines in students’ perception of research importance before and after taking the EIP course.

The Clinical Supervisor’s survey was undertaken after the faculty had received EIP training [21]. This training utilized the online EIP modules [22] developed by NWHSU and UMN through their R25 grant, and in-service trainings at department and general faculty meetings. Although 97% of the clinical supervisors undertook the EIP training, 65% only completed 4 of the 10 modules, which was the minimum EIP training requirement. Outcomes of the survey indicated low to moderate levels of engagement of EIP in the College clinic. More frequent EIP activities were providing treatment references to students, asking students to compare and contrast different approaches to treatment, and identifing sources of information, all of which could have been in relation to East Asian medical information sources. The new electronic resources that were designed to improve information access in the College clinic were used by supervisors and students in about half of their clinic shifts, and may also have been to mainly access East Asian medical information.

The preferential use of East Asian medicine literature sources to inform clinical practice may have been due to insufficient EIP training. However, other explanations are also worthy of consideration. A bias towards greater reliance on East Asian medicine sources would be expected because students and faculty are trained in this discipline. During the thirteen one-hour meetings of the EIP Curriculum Committee, which occurred over a 2.5-year period, much discussion occurred around the meaning of the word evidence [21]. The committee felt strongly that evidence needed to be interpreted broadly, and that East Asian medicine textbooks were an important resource for justifying treatment approaches. Preference for these sources is significantly magnified by the challenges associated with applying the scientific research due to the model validity issues discussed above. However, it is possible that even with research that very accurately resembles real-world practice there will always be a preference for East Asian literature sources. Previous studies of ours [18, 26] and others [39] have shown that faculty and practitioners are concerned about power imbalances between TCIM and biomedicine, and the possibility of cooptation. The validity and historical underpinnings of such issues have been widely discussed in the literature [2, 3]. These are additional and powerful attitudes that could sustain preference for East Asian medical sources.

The significant and consistent increase in students’ self-assessed competence suggested that their EIP skills and knowledge improved as a result of taking the Foundations of EIP course. The development of this course was based on a very similar course that had previously been developed at NWHSU and was shared with Pacific College as part of the collaboration for the NIH grant. Similar improvements in NWHSU’s students EIP skills and knowledge as a result of completing the Foundations of EIP course have been reported [33]. This strongly suggests that this course was useful for imparting EIP knowledge and skills, and that this was sustained across different institutions and instructors. Similar increases in students’ EIP knowledge following the completion of EBM courses have been reported by the other NIH R25 grant recipients [19, 32].

At both NWHSU and Pacific College increases in EIP behaviors were also seen after completing this course. However, these studies don’t indicate the extent to which this translated into an increase in EIP-related activities in the college clinics as a result of this training. Previous studies have shown that successful EBM training does not usually change EBM attitudes and behaviors for both TCIM [4, 32, 40] and biomedical practitioners [41]. Behavioral change models suggest that motivation to adopt EBM is needed along with EBM education and the provision of resources [42], and therefore it is likely that different approaches will be needed to engender greater EIP clinical activity. Additional formative assessment is needed to determine the impact of the EIP training upon faculty and students’ adoption of EIP in the Clinic, and to identify barriers associated with skills, knowledge, availability of resources and time, attitudes and motivation. Such studies may also shed light on how to develop more effective strategies to engender greater use of EIP in clinical activities, some of which may be those developed in the previous NIH R25 grants [19].

This study has limitations. Being an observational study any improvements in EIP skills, knowledge, attitudes and behaviors cannot be conclusively attributed to the EIP training. Due to the inconsistency of answers to the three coding questions in the pre- and post-EIP course surveys we were only able to match 466 of the 1181 completed surveys. The response rate to the open-ended question was 14% for the pre-course surveys and 23% for the post-course surveys, and opinions expressed may not accurately represent all students’ perspectives. The surveys used in this study were not psychometrically tested. The Clinical Supervisors’ survey was administered by the Academic Dean and thus faculty may have felt obligated to respond in a favorable manner. The response rate for this survey was 49% for the pre- and 76% for the post-survey, and may not accurately represent all faculty’s responses. We did not include coding questions in the Clinical Supervisors’ survey and were unable to match pre- and post-respondents. Given that research training is not an accreditation requirement for East Asian medicine programs at the master’s degree level, the outcomes at Pacific College may not be typical for all East Asian medicine colleges in the US. All self-reporting has inherent inaccuracy, and previous studies have shown that EBM surveys are sometimes associated with inaccurately elevated self-reported EBM literacy [43].

## Conclusions

Our study demonstrated that East Asian medicine students recognize the importance of EIP, and that a three-credit EIP course may be effective at improving EIP skills, knowledge and behaviors. Training clinical supervisors and providing greater access to evidence sources in the College clinic did not appear to increase EIP instructional activity. Therefore, it is likely that different approaches are required to improve implementation of EIP in clinical settings.

## Data Availability

The datasets used and/or analyzed during the current study are available from the corresponding author on reasonable request.

## Abbreviations

BA: Belinda Anderson
CIH: Complementary and integrative health
EBM: Evidence-based medicine
Einstein: Albert Einstein College of Medicine
EIP: Evidence informed practice
MYMOP: Measure yourself medical outcome profile
NCCIH: National Center for Complementary and Integrative Health
NIH: National Institutes of Health
NWHSU: Northwestern Health Sciences University
Pacific College: Pacific College of Health and Science
PCOM: Pacific College of Oriental Medicine
SD: Sai Dudla
TCIM: Traditional, complementary and integrative medicine
UMN: University of Minnesota
